# High mortality during tuberculosis treatment does not indicate long diagnostic delays in Vietnam: a cohort study

**DOI:** 10.1186/1471-2458-7-210

**Published:** 2007-08-16

**Authors:** Marleen Vree, Nguyen T Huong, Bui D Duong, Nguyen V Co, Dinh N Sy, Frank G Cobelens, Martien W Borgdorff

**Affiliations:** 1 Research unit, KNCV Tuberculosis Foundation, PO Box 146, 2501 CC, The Hague, The Netherlands; 2 Center for Infection and Immunity Amsterdam (CINIMA), Academic Medical Center, Amsterdam, The Netherlands; 3 National Tuberculosis Programme Vietnam, 463 Hoang Hoa Tham street. Ba Dinh District, Hanoi, Vietnam; 4 KNCV Tuberculosis Foundation, PO Box 146, 2501 CC, The Hague, The Netherlands

## Abstract

**Background:**

Delay in tuberculosis diagnosis and treatment initiation may increase disease severity and mortality. In evaluations of tuberculosis control programmes high fatality rates during tuberculosis treatment, are used as an indicator of long delays in low HIV-prevalence settings. However, data for this presumed association between delay and fatality are lacking. We assessed the association between diagnostic delay and mortality of new smear-positive pulmonary tuberculosis patients in Vietnam.

**Methods:**

Follow-up of a patient cohort included in a survey of diagnostic delay in 70 randomly selected districts. Data on diagnosis and treatment were extracted from routine registers. Patients who had died during the course of treatment were compared to those with reported cure, completed treatment or failure (survivors).

**Results:**

Complete data were available for 1881/2093 (89.9%) patients, of whom 82 (4.4%) had died. Fatality was 4.5% for patients with ≤ 4 weeks delay, 5.0% for 5- ≤ 8 weeks delay (aOR 1.11, 95%CI 0.67–1.84) and 3.2% for > 9 weeks delay (aOR 0.69, 95%CI 0.37–1.30). Fatality tended to decline with increasing delay but this was not significant. Fatality was not associated with median diagnostic delay at district level (Spearman's rho = -0.08, P = 0.5).

**Conclusion:**

Diagnostic delay is not associated with treatment mortality in Vietnam at individual nor district level, suggesting that high case fatality should not be used as an indicator of long diagnostic delay in national tuberculosis programmes.

## Background

Worldwide around 2 million people die with tuberculosis each year [1]. Of all new smear-positive pulmonary tuberculosis patients treated in programmes applying the World Health Organization's (WHO) DOTS strategy, 4.6% died [1].

It is generally assumed that delay in diagnosis and treatment initiation increases the severity of disease at the time of diagnosis and the risk of death [2,3]. This assumed relation is often reason to measure the length of delays before tuberculosis treatment [4-6]. Conversely, a high case fatality, i.e. death during the course of tuberculosis treatment, is often perceived to reflect long delays in low HIV-prevalence settings. In program evaluations high case fatality rates are thereby used as an indicator of low access to clinics [7].

With time, tuberculosis disease progresses [8] and chances of survival will decline. With worsening and spreading of pulmonary tuberculosis lesions the bacillary load in the lungs generally increases [8]. Therefore, longer delays could lead to more progressed disease with higher bacillary loads in sputum, which could result in higher fatality.

Delayed treatment was strongly associated with death in a study carried out in Canada [9], and with death among HIV-positive patients, but not among HIV-negative patients, in a study from New York City [10]. Delay in seeking treatment was not an independent predictor for tuberculosis mortality during on average 2.3 year period after diagnosis in Mexico [11]. A study from South Africa showed that those who survived had a significant shorter median treatment delay than those who had died [12]. However, this study used treatment delay instead of total delay. Treatment delay is the time period from first seeking help for symptoms until treatment initiation. Therefore, treatment delay is a fraction of the total diagnostic delay, i.e. the time period from onset of tuberculosis symptoms to treatment initiation. Therefore, total diagnostic delay is possibly a better indicator of the duration of illness than treatment delay. In high-burden tuberculosis settings, no studies have been carried out on the association of total diagnostic delay with tuberculosis mortality.

Vietnam has a high burden of tuberculosis, with 58,389 new smear-positive tuberculosis patients notified in 2004 (70/100,000 population) [1]. The DOTS strategy has been implemented nationwide since 2000 [13]. Treatment outcomes of 90% cure and 3.4% death for new smear-positive tuberculosis cases in 2002 were reported by the National Tuberculosis Control Programme Vietnam (NTP) [14]. The prevalence of HIV infection in the adult population was 0.5% in 2005 (range 0.3–0.9%) [15]. Mean diagnostic delay was 7 weeks and 15% of patients had reported a delay of 12 weeks or more in 2002 [16].

As data for the presumed association between delay and fatality are lacking, it is unknown whether fatality can be used as an indicator for long delays in programme evaluations. This study aims to assess the association between total delay and fatality of new smear-positive pulmonary tuberculosis patients in a programmatic ambulant setting in Vietnam.

## Methods

A cohort of 2,093 patients with newly diagnosed smear-positive tuberculosis was followed who were included in a survey of diagnostic delay carried out in 2002. That study included all patients consecutively registered for treatment over a period of 3 months in 70 randomly selected districts in Vietnam. Study population and methods are described elsewhere [16]. Diagnostic delay was defined as the period between the onset of cough and treatment initiation. Patients were interviewed using a pre-coded structured questionnaire including demographic variables; time period between onset of cough and treatment initiation; date of diagnosis; starting date of treatment. The district tuberculosis coordinators were trained on performing interviews and interviewed patients within 2 weeks of treatment registration.

Case definitions of the NTP follow international recommendations [17].

Included were patients aged ≥ 15 years, registered as new smear-positive pulmonary tuberculosis with registered treatment outcome of cure, treatment completion, failure or death following treatment with the standard NTP regimen of short-course chemotherapy (SCC) for new smear-positive pulmonary tuberculosis. This regimen contains daily streptomycin, isoniazid, rifampicin and pyrazinamide for 2 months, followed by daily isoniazid and ethambutol for 6 months (2SHRZ/6HE). To avoid misclassification, we excluded patients with an unknown survival status, i.e. patients with a registered treatment outcome of default or transfer-out or unevaluated treatment outcome.

An indicator of the bacillary load in the sputum is the sputum grade at diagnosis [18], which is a grading of the reported number of acid-fast bacilli found in a defined number of immersion fields of a Ziehl-Neelsen stained sputum smear [19]. The NTP has a standard recording and reporting system for smear examination [20]. Classification of sputum grade of the NTP follows international recommendations [21]. The NTP's laboratory quality control system reports 99% concordance between smear reading in the tuberculosis clinics and rereading in provincial laboratories [22].

Data on diagnosis, treatment monitoring and outcome were extracted from routine laboratory and treatment registers. A pre-coded and pre-tested structured form included information on date of treatment initiation, treatment regimen, treatment outcome, date of treatment completion or death and Ziehl-Neelsen stain grade at diagnosis and at 2, 5 and 8 months. Blinded to the original forms, information on 10% of randomly selected patients was double collected from routine registers and checked for inconsistencies (1.7% of items).

The research board of the National Hospital for Tuberculosis and Respiratory Diseases, Hanoi gave scientific and ethical clearance for the study.

Death as treatment outcome or fatality is defined as death due to any cause during the course of treatment [17]. Death directly due to tuberculosis most likely occurs during the first months of treatment [23] and therefore early mortality was defined as a reported death within 3 months after treatment initiation.

Data were entered using Epi Info 2002 and a 10% randomly selected sample of forms were re-entered in blinded fashion, resulting in inconsistencies of data entry in 0.22% of items. Analyses were performed using Stata/SE V8.0 (Stata Corp., College Station TX, USA).

The analysis was restricted to patients with known vital status at the end of treatment. Survival was defined as a reported treatment outcome of cure, treatment completion or treatment failure. Survival cases were compared to patients with reported death. The sputum grade at diagnosis was calculated as the highest reported sputum grade observed in the examined smear slides.

Survival time was calculated as the time interval from date of treatment initiation to the date of treatment outcome. Survival curves up to 4 months after treatment initiation were constructed for three classes of diagnostic delay using the Kaplan-Meier method.

As diagnostic delay as a continuous variable was not normally distributed, it was transformed by taking its natural logarithm (Ln).

To assess differences at the 5% significance level, two-sided Fisher's exact test or Chi-square tests were used.

Primary analyses included logistic regression on fatality during the total course of tuberculosis treatment. The multivariate model was built as follows. Apart from diagnostic delay, age was thought to be associated with fatality and thus included in the multivariate model. Other variables and interaction terms were included if the term contributed to the model with a p value < 0.10. If the p-value of a covariate or interaction term was < 0.1 and confounded the association between delay and fatality, we decided it contributed to the model, and it was included in the final model.

Secondary analyses included survival analyses on early fatality. The risk of early fatality was compared between subgroups to identify risk factors. Cox proportional hazard modelling was used for adjustment of confounders.

Correlation on district level between mean and median diagnostic delay and fatality was analysed using Spearman's rho. Fatality was the proportion of deaths of all who started treatment in a district in 2002 and 2003 as reported by the quarterly reports of that district.

## Results

**Figure 1:**
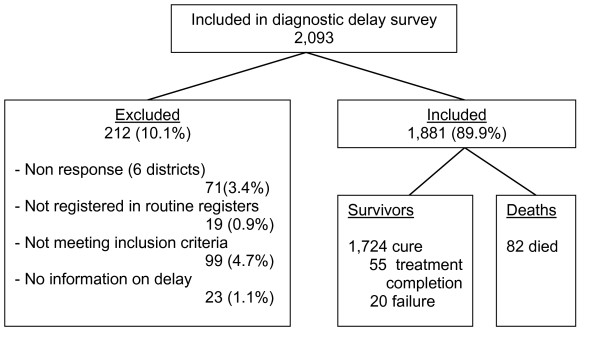
Inclusion of patients.

Of 2,093 eligible patients, this study included 1,881 patients (89.9%) of whom 82 (4.4%, 95%CI 3.5–5.4%) had died (Figure 1).

**Figure 2:**
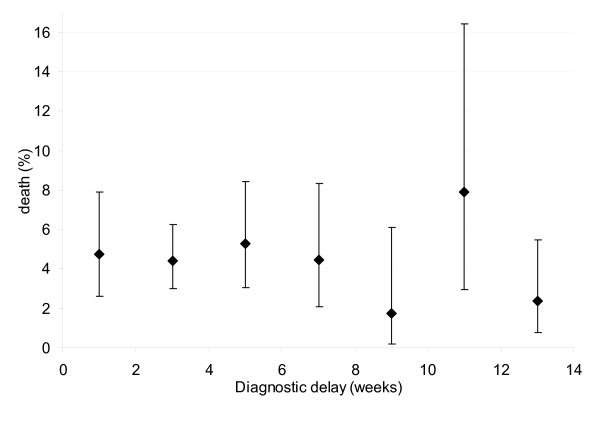
Proportion mortality with 95%CI during treatment by 2-week classes of diagnostic delay.

**Figure 3:**
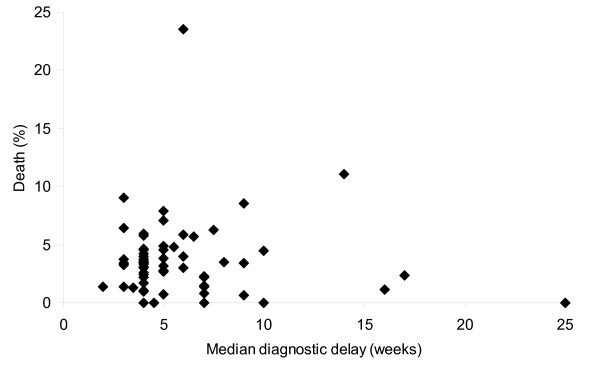
Mean fatality in 2002–2003 and median diagnostic delay per district. Each data point represents one district.

Among excluded subjects male sex and older age were more common than among included subjects (Table 1). Characteristics of patients by survival status are described in Table 2. Mean and median delay were 5.6 (SD 4.5) and 4 weeks (inter-quartile range (IQR) 3–7) for patients who died. These were 7.2 (SD 9.1) and 4 weeks (IQR 3–8) for patients who survived. Fatality did not increase with longer diagnostic delay at the individual level (Figure 2). At the level of districts, fatality did not correlate with mean or median diagnostic delay (Spearman's rho = -0,08, P = 0.5) (Figure 3).

**Table 1:** Characteristics of in- and excluded subjects in the study

	All	Included N (%)	Excluded N (%)	p
All	2093	1881 (89.9%)	212 (10.1%)	
Diagnostic Delay				0.196
≤ 4 weeks	1059	975 (92.1%)	84 (7.9%)	
5- ≤ 8 weeks	561	504 (89.8%)	57 (10.2%)	
> 9 weeks	448	402 (89.7%)	46 (10.3%)	
missing	25	0	25	
Sex				0.0038
Men	1491	1326 (88.9%)	165 (11.1%)	
Women	596	555 (93.1%)	41 (6.9%)	
Age, years				0.006
15–34	515	490 (95.1%)	25 (4.9%)	
35–54	823	753 (91.5%)	70 (8.5%)	
≥ 55	715	638 (89.2%)	77 (10.8%)	

**Table 2:** Survival status by risk factors among new smear-positive tuberculosis patients

	Survival N	Death N (%)	P value	Crude OR (95%CI)	Adjusted* OR (95%CI)
All (n = 1,881)	1799	82 (4.4%)			
Diagnostic Delay			0.42		
Continuous†, per Ln(delay-week)	1799	82 (4.4%)		0.77 (0.57–1.03)	0.78 (0.58–1.05)
≤ 4 weeks	931	44 (4.5%)		1	1
5- ≤ 8 weeks	479	25 (5.0%)		1.10 (0.67–1.83)	1.11 (0.67–1.84)
> 9 weeks	389	13 (3.2%)		0.71 (0.38–1.33)	0.69 (0.37–1.30)
Sex			0.138		
Men	1262	64 (4.8%)		1	1
Women	537	18 (3.2%)		0.66 (0.39–1.13)	0.58 (0.34–1.00)
Age, years			0.010		
15–34	470	20 (4.1%)		1	1
35–54	731	22 (2.9%)		0.71 (0.38–1.31)	0.68 (0.37–1.27)
≥ 55	598	40 (6.3%)		1.57 (0.91–2.7)	1.64 (0.37–1.27)
Sputum grade at diagnosis (n = 1,827)			0.256		
Scanty	119	10 (7.8%)		2.1 (1.01–4.2)	2.1 (1.02–4.3)
1+	1028	42 (3.9%)		1	1
2+	370	18 (4.6%)		1.19 (0.68–2.1)	1.22 (0.69–2.2)
3+	230	7 (4.2%)		1.06 (0.53–2.2)	1.14 (0.56–2.3)

* Adjusted for age group, diagnostic delay and sex

OR = Odds Ratio

† Log transformation

Fatality declined, but not significantly, with increasing delay in the crude and adjusted analyses (aOdds Ratio (OR) 0.78 per log delay-week) (Table 2). Fatality was lowest with 3.2% for those with diagnostic delays > 9 weeks, but did not significantly differ from delay ≤ 4 weeks (aOR 0.69) (Table 2).

In Vietnam HIV prevalence is highest among young adults and reported long delays may be most prone to recall bias. Results were very similar for the crude and adjusted OR for delay as a continuous and as a categorical variable after excluding patients with extreme delays of ≥ 27 weeks or patients aged < 35 years from the analyses. Therefore, young patients and extreme delays did not explain the lack of an expected increase in fatality with diagnostic delay. We therefore included these subjects in the analysis.

**Figure 4:**
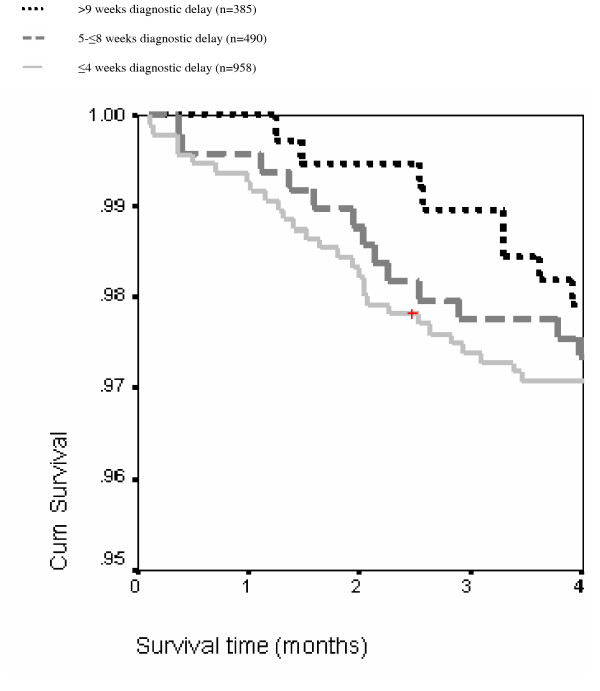
Kaplan-Meier survival curve for the first 4 months after treatment initiation for 1,759 surviving and 74 deceased patients by duration of diagnostic delay.

Of 82 deaths, 40 occurred ≤ 3 months after treatment initiation and had available data on survival time (2.2%, 95%CI 1.6–3.0%). Survival was not higher for those with delay > 9 weeks compared to those with delay ≤ 8 weeks (HR 0.42, 95%CI 0.15–1.17) (Figure 4). The unadjusted hazard for early mortality declined significantly with 0.62 (95%CI, 0.41–0.96) per Ln(delay-week). This was not affected by adjustment for age, sex or sputum grade. The hazard ratio (HR) was lowest with 0.40 for those with delay > 9 weeks. The difference with delay ≤ 4 weeks was not significant.

Sputum grade at diagnosis may be on the causal pathway between diagnostic delay and death, and we tested these associations. Sputum grade at diagnosis differed significantly by diagnostic delay (P = 0.008). However, no clear pattern was observed in the association of sputum grades with diagnostic delay (data not shown). A scanty sputum grade at diagnosis was significantly associated with fatality (aOR 2.1), but higher sputum grades at diagnosis were not (Table 2). Results for the association of diagnostic delay with fatality were very similar after adjustment for sputum grade at diagnosis (data not shown).

## Discussion

We hypothesized that high mortality rates would indicate long diagnostic delay. This study showed that mortality during tuberculosis treatment did not increase with diagnostic delay on individual and district level; if anything, there was a trend towards shorter delay. This association was not explained by age, sex or sputum grade at diagnosis.

Death as a result of tuberculosis pathology is most likely to occur during the first months after treatment initiation. During the initial phase of treatment (2 or 3 months) a rapid stop is put to bacterial multiplication, which limits further deterioration and death after the first weeks of treatment initiation [23]. The numbers of bacilli are low in the continuation phase [24] and causes of death other than tuberculosis play a greater role. However, early fatality did also not increase with diagnostic delay.

These study results do not confirm findings from Canada [9]. Possible explanations are differences in progression rates between patients or in influence of co-morbidity on progression rates and mortality, or biased results due to study limitations.

Progression from infection to tuberculosis disease as well as the severity of disease may differ between subjects. Increasing delay may lead to increasing mortality if disease progresses in a similar way across the total study population. However, some patients may have rapid disease progression and in these patients short delay may be associated with high mortality. Patients with rapid progression cannot be differentiated from patients with slow progression in this study nor in programmatic settings. Possibly the proportion of patients with rapid progression outweighs the proportion of patients with regular or slow progression of tuberculosis. Differing progression rates between patients implies that mortality cannot be used as an indicator of long delays.

The prevalence of co-morbidity may differ by setting. Tuberculosis patient may suffer simultaneously from other diseases, such as malignancies, diabetes or HIV which can affect the development of tuberculosis and mortality both [25]. For instance, HIV-infection is a strong risk factor for death during tuberculosis treatment [26,27] and HIV-infection increases the rate of progression from infection to disease [26]. Therefore, co-morbidity may have modified the effect of delay on mortality. Co-morbidity as a risk factor for development of tuberculosis is probably more common among patients in a low incidence tuberculosis setting or in a high HIV-prevalence setting. For instance, the estimated prevalence of diabetes was with 7.5% in Canada higher than in Vietnam with 2.3% in 2000 [28]. Likely, co-morbidity has influenced the differences in study findings from Vietnam compared with Canada [9]. In Vietnam, the HIV-prevalence was low during the study period [15]. More than 60% of HIV-infections occurred among people aged < 35 [29]. However, after excluding tuberculosis patients aged < 35 years associations were very similar. Therefore, our findings are not representative for settings with high HIV prevalence.

Higher sputum grade at diagnosis was associated with higher mortality in India [30], but not in this study. Subjects with a sputum smear graded as scanty may have been immune compromised and therefore may have higher risk of mortality. However, scanty sputum smear results did not explain the tendency towards a declining trend of mortality with longer delays.

Another explanation for the lack of association between diagnostic delay and fatality may be the short diagnostic delay in Vietnam as compared to other countries [16]. Possibly, tuberculosis fatality is increased if disease duration has been sufficient for cavity formation with associated lung damage, and chemotherapy cannot overcome this damage anymore. The time needed for such severe damage may be much longer than 9 weeks. Whether high fatality can be used as indicator for long diagnostic delays in settings with long delays, needs investigation.

The study limitations include patient selection and data validity. Amongst the excluded patients were patients registered with treatment default or transfer-out. Some of these patients could have had died within 8 months, i.e. the duration of chemotherapy [31]. If all these patients had died, fatality would have been 74 (7.7%) for those with delay ≤ 4 weeks, 35 (7.2%) for those with 4- ≤ 8 weeks and 29 (7.2%) for those with > 9 weeks delay. Therefore, it is unlikely that this seriously biased the results.

Case fatality rates were based on registered treatment outcomes and deaths could have been underreported. A follow-up study in northern Vietnam on survival and relapse or patients with a registered treatment outcome of cure or treatment completion did not reveal underreporting of death [32]. It was therefore concluded that treatment success was accurately recorded. Even if some cured patients were in fact treatment failures, then in our analyses, both cured and failures were analysed as survivors and would therefore not have biased the results. Furthermore, it is unlikely that reporting of death depends on delay. If death was underreported, it is probably non-differential for delay and it would not have biased the results.

No gold standard exists for valid assessment of diagnostic delay. The duration of illness as recalled by the patient may not be accurate, and misclassification in delay categories can have occurred. This would have resulted in a diluted association between diagnostic delay and mortality. Especially among those who reported extreme delays, recall bias may have played a role. However, extreme delays did not explain the observed trend towards declining mortality with longer delays.

Our findings cannot be extrapolated to patient groups who were not included in the study. These groups are patients who were diagnosed with tuberculosis but did not start treatment, and patients treated in provincial and national tuberculosis hospitals. This does not undermine the importance of our findings, as in programme evaluations the indicator of case fatality is not used on (non-existent) data of patients who did not start treatment.

Our findings cannot be applied to settings with high HIV prevalence or high co-morbidity among tuberculosis patients, such as in low incidence tuberculosis countries. However, in these settings high case fatality does probably not reflect long delays. Our findings imply that in routine practice in settings with a high burden of tuberculosis a high case fatality does also not reflect long diagnostic delays.

## Conclusion

Diagnostic delay is not associated with mortality during tuberculosis treatment in ambulant programmatic settings in Vietnam. This suggests that for such settings high case fatality should not be used as an indicator of long diagnostic delay.

## Competing interests

The author(s) declare that they have no competing interests.

## Authors' contributions

MV and MB are responsible for the conception of the study and all authors are responsible for the study design. MV and NH contributed to the acquisition of the data. MV planned and conducted the study and drafted the first version of the manuscript. MV, FC and MB contributed to analysis and interpretation of data. All authors have been involved in revising it critically for important intellectual content. All authors read and approved the final manuscript.

## Pre-publication history

The pre-publication history for this paper can be accessed here:


